# Electroencephalogram of Normotensive Individuals During Sitting and Standing Blood Pressure Measurement Positions

**DOI:** 10.7759/cureus.72154

**Published:** 2024-10-22

**Authors:** Mayowa J Adeniyi, Ayobami A Adamu, Ayoola Awosika

**Affiliations:** 1 Department of Physiology, Federal University of Health Sciences, Otukpo, NGA; 2 Department of Anesthesia, Ekiti State University Teaching Hospital, Ado-Ekiti, NGA; 3 College of Medicine, University of Illinois, Chicago, USA

**Keywords:** alpha waves, beta wave, eeg (electroencephalogram), powerlab, sphygmomanometer

## Abstract

Background

Investigating changes in brain electroencephalogram activity during blood pressure measurement in sitting and standing positions is clinically significant for understanding the neural correlates of postural changes, which may affect cerebral perfusion and autonomic regulation. Shifts in blood pressure can influence visual cortical activity, potentially altering cognitive and sensory processing. This research holds clinical relevance in evaluating disorders like orthostatic hypotension and syncope, where postural changes compromise cerebral blood flow. Furthermore, it can help refine diagnostic protocols and management strategies for neurovascular and autonomic dysfunction. The study also highlighted the possibility of using polygraphs to identify and manage discrepancies in blood pressure measurement caused by posture-induced changes in brain activities for accurate diagnosis.

Methodology

The electroencephalogram of healthy normotensive female individuals undergoing blood pressure measurements at sitting and standing positions was investigated. Ten healthy young adult females who satisfy the inclusion criteria were selected. Electroencephalographic (EEG) waves were recorded with the aid of Powerlab 26T (ADInstruments Pty Ltd., Bella Vista, Australia). Blood pressure and pulse rate measurements were conducted using a sphygmomanometer and stopwatch.

Results

During sitting and standing blood pressure positions, diastolic blood pressure and pulse rate were higher when compared to baseline values but the standing blood pressure position showed a higher pulse rate than the sitting blood pressure position. Although alpha wave frequency was higher during both sitting and standing blood pressure measurement positions, the standing blood pressure position caused lower alpha wave frequency when compared with the sitting blood pressure measurement position. While a strong negative correlation was found between alpha wave frequency and pulse rate, beta wave frequency positively correlated with pulse rate during sitting blood pressure measurement position. Furthermore, during standing blood pressure measurement position, the alpha wave did not correlate with pulse rate nor was there a correlation between beta wave frequency and pulse rate. In conclusion, the present study indicated that both sitting and standing blood pressure measurement positions caused a decrease in alpha wave frequency when compared with baseline, but the standing blood pressure measurement position elicited a lower alpha wave frequency when compared with the sitting blood pressure measurement position.

## Introduction

Blood pressure is the force within the cardiovascular circuit required to maintain blood circulation. Deviations from normal blood pressure indicate changes in metabolic demands and health challenges [1,2]. Hypertension, a sustained increase in blood pressure, is an asymptomatic disease that affects thousands of people worldwide. Regular blood pressure monitoring is one of the major viable ways of early detection of the condition. The invention of the noninvasive blood pressure measurement technique by Nikolai Korotkoff in 1905 has significantly enabled prompt detection of the level of tension in the cardiovascular system [1-3]. Although non-invasive blood pressure measurement using the auscultatory or palpatory method is a painless procedure, it requires the application of external pressure to compress and occlude a peripheral artery temporarily. This causes a perception of pressure and activation of brain areas responsible for pressure perception.

There is a plethora of evidence to suggest that changes occur in brain activities during blood pressure monitoring in healthy subjects. Davies et al. reported that ambulatory blood pressure measurements elicited arousal from sleep in 10 healthy individuals [4]. In a study by Socrates et al., the impact of measuring blood pressure using a cuff inflation-related procedure was investigated [5]. There was more arousal with cuff inflation measurements when compared with cuffless measurements. This study indicated that in addition to tactile stimuli and pressure, pump noise created by cuff inflation could alter the quality and duration of sleep and brain activity [6,7].

Furthermore, in healthy subjects, cuff inflation measured every hour was reported to be associated with increased arousal and wakefulness [8]. In 234 healthy adolescents, Lehrer et al. indicated that cuff inflation during ambulatory blood pressure measurement temporarily increased the restlessness of the subjects during sleep [9]. Gaffey et al. reported that ambulatory blood pressure monitoring using cuff-related blood pressure caused a disturbance in sleep among healthy adults [10]. Conversely, Sherwood et al. reported that ambulatory blood pressure measurement exerted no significant influence on sleep quality in people, especially African-Americans with untreated hypertension [11].

Electroencephalogram, as a non-invasive technique, makes understanding of brain activities easy under different physiological situations. For instance, Arousal, wakefulness, and sleeplessness reported with blood pressure monitoring in healthy individuals are associated with changes in beta wave frequency. In previous studies, prolonged unipedal orthostasis, post-exercise orthostasis, sudden sit-stand switch, and rotatory chair activity were shown to alter electroencephalogram in apparently healthy individuals [7,12,13]. The study aimed to record the electroencephalogram of normotensive individuals at sitting and standing blood pressure monitoring positions.

## Materials and methods

The research was conducted in the Technologically Enhanced Laboratory Unit of the Department of Physiology, College of Medical Sciences, Edo State University Uzairue, situated in Etsako West Local Government Area of Edo State, Nigeria. A similar study has neither been done in disease patients nor healthy individuals.

Study design

Twenty-five young female individuals who were third-year medical students of Edo State University Uzairue were recruited for the study through respondent-driven sampling. Only 10 healthy young adult females who satisfied the inclusion criteria were selected. The average age of the participants was 19.6 years (range of 17-21 years). Written consent was obtained from each participant.

Inclusion criteria

Female participants aged 17 to 21 years were recruited for the study. Other inclusion criteria were based on blood pressure, pulse rate, and respiratory rate of participants, with a cut-off set of the following range: systolic blood pressure of 90-120 mmHg; diastolic blood pressure of 60-80 mmHg; pulse rate of 60-100 BPM; and respiratory rate of 12-20 cycles/minute. 

Exclusion criteria

A well-structured questionnaire was administered to rule out those with a medical history of smoking and caffeine and any form of medication, kidney, hepatic, and metabolic diseases as previously reported [12,14]. Participants with a history of respiratory and cardiovascular diseases or those whose blood pressure, pulse rate, and respiratory rate are higher or lower than normal range were exempted. Those with anatomical deformities were also not accommodated into the study.

Measurement of electroencephalographic (EEG) waves

EEG waves were recorded with the aid of Powerlab 26T (ADInstruments Pty Ltd., Bella Vista, Australia) for two minutes (time taken for inflation and deflation of BP cuff). As indicated in the manual, both white and blue marked electrodes were connected to the left and right side of the frontal part of the skull, while the black electrode was attached to the occiput. Electrodes were held in place by means of electrode pads. As part of the measures aimed at preventing artifacts, ambient noise interference was avoided.

In a sitting position without blood pressure measurement, a baseline electroencephalogram was done. With electrodes attached, an electroencephalogram was recorded during sitting and standing blood pressure measurement positions.

The alpha/beta ratio was calculated by dividing alpha wave frequency by beta wave frequency.

Some of the key overarching objectives of this study were to record alpha waves during sitting and standing blood pressure measurement positions; record beta waves during sitting and standing blood pressure measurement positions; determine alpha/beta ratio during sitting and standing blood pressure measurement positions; determine whether there is an association between cardiovascular parameters and EEG waves during sitting and standing blood pressure measurement positions.

Measurement of blood pressure and pulse rate

Blood pressure was measured from the arm, an inch above the elbow, using a Mercurial Sphygmomanometer (Kris Aloy, Shanghai SNWI MEDICAL Co., Ltd.). Baseline blood pressure values were recorded in a sitting position for all participants at the start of the study. The time required for inflation and deflation of the inflatable cuff was noted. 

Baseline readings were taken at a sitting position without EEG electrodes attached, as previously reported [15-17].

Blood pressure was also measured both in sitting and standing positions with EEG electrodes attached to the scalp, and an electroencephalogram was conducted.

Pulse rate was determined as the number of arterial pulsations in one minute. Baseline readings were taken at a sitting position without EEG electrodes attached.

Pulse rate was also determined both in sitting and standing positions, with EEG electrodes attached to the scalp, and an electroencephalogram was conducted.

Pulse pressure was determined by subtracting diastolic blood pressure from systolic blood pressure.

Statistical analysis

Statistical analysis was conducted using Statistical Package for Social Science Students (SPSS) (IBM SPSS Statistics for Windows, IBM Corp., Version 23, Armonk, NY). A statistical test was done using analysis of variance (ANOVA), and a correlational analysis was performed using Pearson correlation. A statistically significant difference was accepted at P < 0.05.

## Results

Effect of blood pressure measurement on cardiovascular parameters during sitting and standing positions

Table 1 shows that systolic blood pressure did not significantly change during sitting and standing blood pressure measurement positions. Diastolic blood pressure was significantly (P < 0.05) increased during the standing blood pressure position. Pulse pressure did not significantly change during sitting and standing blood pressure measurement positions. During the standing blood pressure position, the pulse rate was significantly (P < 0.05) increased.

**Table 1:** Effect of blood pressure measurement on cardiovascular parameters during sitting and standing positions Descriptive statistics: A statistically significant difference was accepted at P < 0.05. *Significant difference from baseline at P < 0.05 ^a^Significant difference from sitting BP position at P < 0.05 BPM: beats per minute; BP: blood pressure; mmHg: millimeter mercury; SEM: standard error of the mean

Parameters	Baseline mean ± SEM	Sitting BP position mean ± SEM	Standing BP position mean ± SEM
Systolic blood pressure (mmHg)	107.0 ± 0.633	107.5 ± 2.371	109.0 ± 2.530
Diastolic blood pressure (mmHg)	74.0 ± 1.265	75.5 ± 0.474	76.5 ± 0.158*
Pulse pressure (mmHg)	33.0 ± 1.897	32.0 ± 1.897	32.5 ± 2.688
Pulse rate (BPM)	78.5 ± 0.158	78.0 ± 0.316	83.0 ± 0.316*^a^

Effect of blood pressure measurement on alpha wave frequency during sitting and standing positions

**Figure 1:**
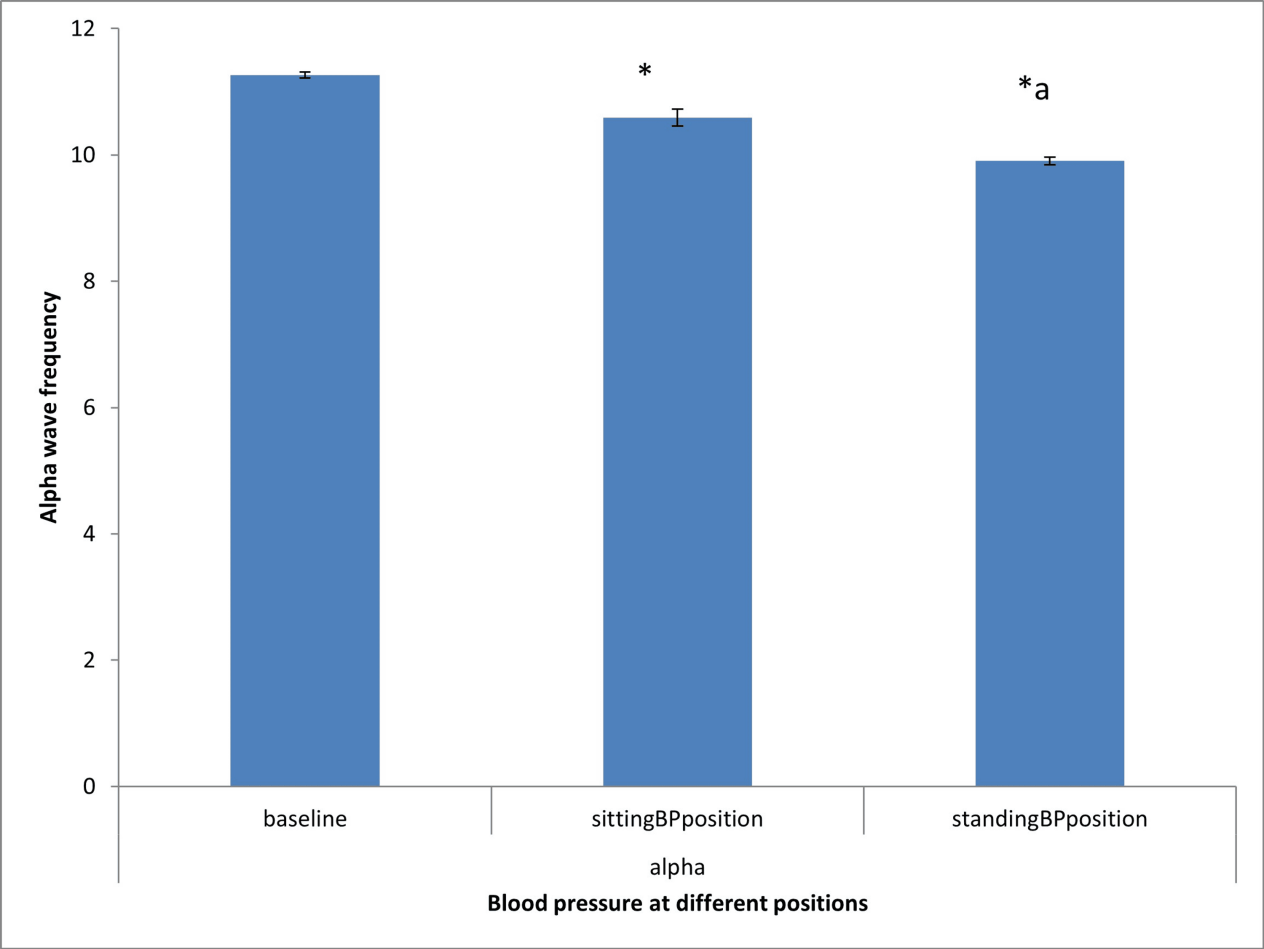
Effect of blood pressure measurement on alpha wave frequency during sitting and standing positions Analysis of variance: A statistically significant difference was accepted at P < 0.05. *Significant difference from sitting blood pressure position at P < 0.05 ^a^Significant difference from standing blood pressure position at P < 0.05 BP: blood pressure

Figure 1 shows that both sitting and standing blood pressure measurement positions significantly (P < 0.05) decreased alpha wave frequency compared to the baseline group. When compared with the sitting blood pressure measurement position, the standing blood pressure measurement position caused a significant decrease in alpha wave frequency.

Effect of blood pressure measurement on beta wave frequency during sitting and standing positions

**Figure 2:**
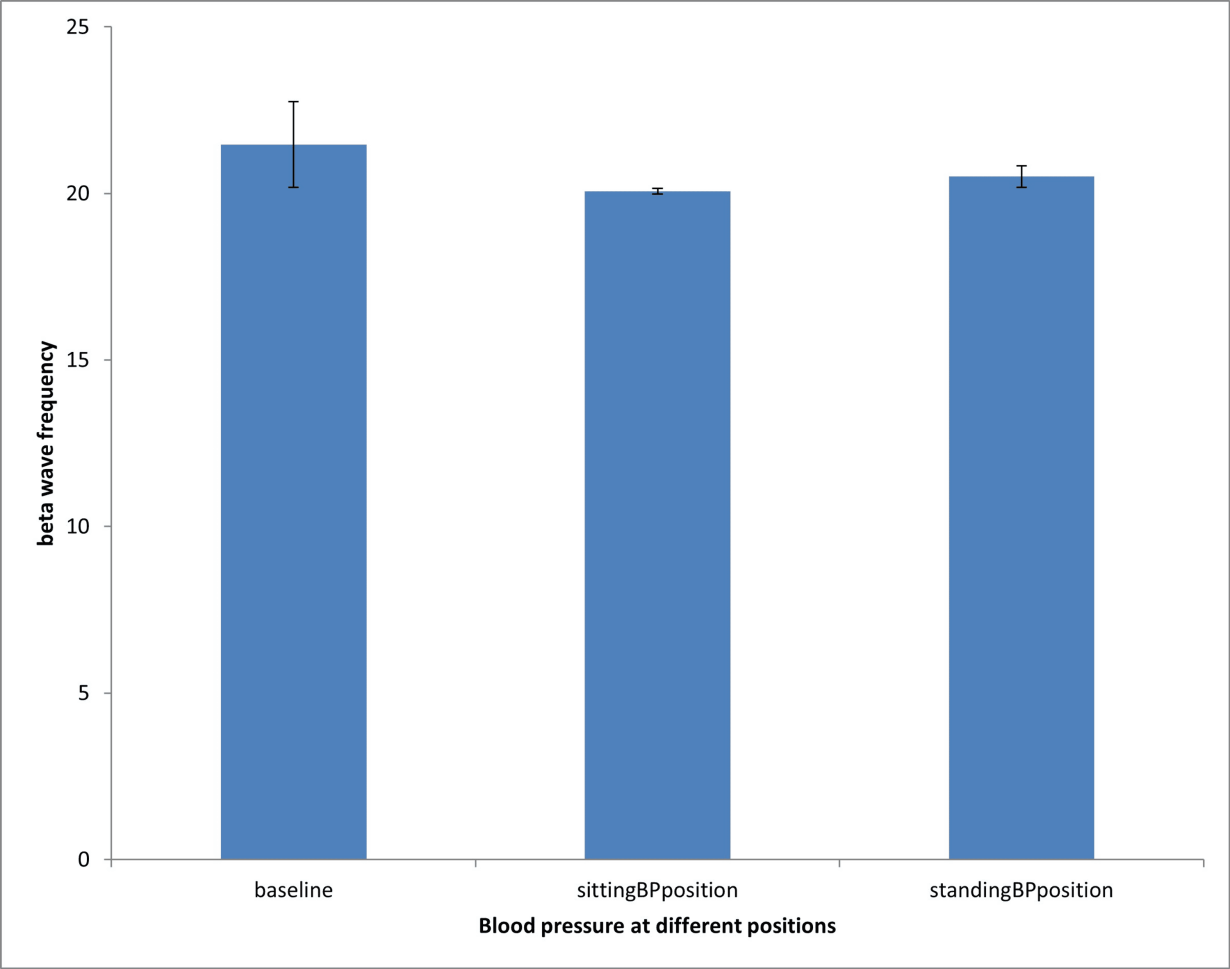
Effect of blood pressure measurement on beta wave frequency during sitting and standing positions Analysis of variance: A statistically significant difference was accepted at P < 0.05. BP: blood pressure

Figure 2 shows that both sitting and standing blood pressure measurement positions did not significantly affect beta wave frequency compared to the baseline group.

Effect of blood pressure measurement on alpha/beta ratio during sitting and standing positions

**Figure 3:**
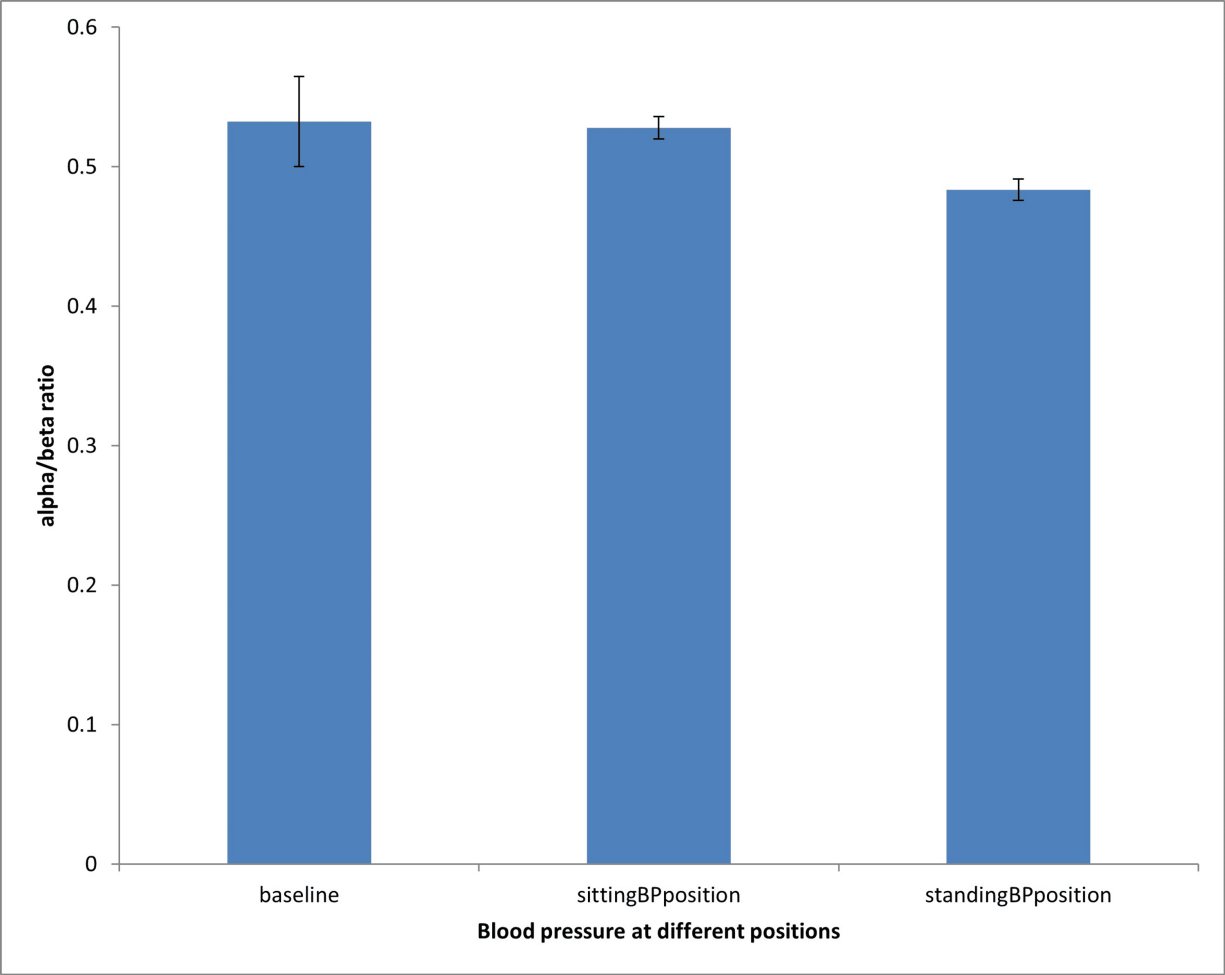
Effect of blood pressure measurement on alpha/beta ratio during sitting and standing positions Analysis of variance: A statistically significant difference was accepted at P < 0.05. BP: blood pressure

Figure 3 shows that both sitting and standing blood pressure measurement positions did not significantly affect the alpha/beta ratio when compared with the baseline group.

Correlational analysis between pulse rate and EEG waves during sitting and standing blood pressure monitoring

Table 2 shows a significantly strong negative correlation between pulse rate and alpha wave frequency during sitting blood pressure measurement. A significant strong positive correlation was observed between pulse rate and beta wave frequency during sitting blood pressure measurement position.

**Table 2:** Correlation between pulse rate and EEG waves during sitting blood pressure monitoring in sitting position Correlation analysis: A statistically significant difference was accepted at P < 0.05 *Significant difference P < 0.05

Correlations	Alpha wave frequency	Beta wave frequency
Pulse rate	-0.816*	0.687*

Table 3 shows an insignificant strong positive correlation between pulse rate and alpha wave frequency during standing blood pressure measurement. There was an insignificant correlation between pulse rate and beta wave frequency during standing blood pressure measurement position.

**Table 3:** Correlation between pulse rate and EEG waves during standing blood pressure monitoring in standing position Correlation analysis: A statistically significant difference was accepted at P < 0.05.

Correlations	Alpha wave frequency	Beta wave frequency
Pulse rate	0.186	0.278

## Discussion

There is mounting evidence that ambulatory blood pressure measurements may interfere with brain activities during sleep, perhaps altering the prominence of high-amplitude waves such as delta waves [4,5,9]. Tactile and mechanical stimulations and noise generated have been identified as potential causes of changes in brain activities during blood pressure measurements during sleep [6,18]. The present study sought to understand whether EEG waves, especially alpha and beta waves, are altered during single routine blood pressure measurements at sitting and standing positions in healthy female individuals.

During standing blood pressure measurement, diastolic blood pressure and pulse rate were higher when compared with baseline. Pulse rates were higher when compared with baseline and sitting blood pressure measurements, respectively. In literature, posture, especially standing, has been documented to cause cardiovascular and hemodynamic changes [9,19,20]. Depletion of central blood volume elicits the inactivation of baroreceptors, leading to sympathetic activation, an increase in blood pressure, an increase in heart rate, and many more [21]. 

The principal finding of the study was that when compared with baseline, there was a decrease in alpha wave frequency during sitting and standing blood pressure measurement positions with alpha wave frequency lowered during the standing position than the sitting position. Conventionally, alpha wave frequency was known to be more prominent during the first stage of sleep and relaxed wakefulness with closed eyes since the waves are produced by spontaneous reticular thalamic discharge imposed on the visual cortex [13,22]. Fatigue and dizziness have also been recognized as influencers of alpha wave frequency. Hence, the decreases in alpha wave frequency observed during sitting blood pressure measurement position signified the metabolic state of the brain during blood pressure measurement. Discrepancies in blood pressure measurement between home and clinic have been widely reported and are majorly due to the inability of patients to observe rest. Since less cumbersome EEG procedures, such as Powerlab, are available that can evaluate blood pressure and electroencephalogram concurrently, the tendency of discrepancy and inaccuracy in blood pressure measurements due to increased brain activities can be recognized and factored out.

The reduction in alpha wave frequency during standing blood pressure position when compared to sitting blood pressure measurement position concurs with the report of previous studies [7,12]. Standing is known to cause the diversion of blood to the lower extremities, resulting in the activation of the sympathetic nervous mechanism and an increase in alertness. During alertness, anxiety, and physical stress, alpha wave frequency and alpha/beta ratio are known to reduce [23-25].

Furthermore, the inverse correlation between alpha wave frequency and pulse rate during sitting blood pressure measurement implies that as alpha wave increases during sitting blood pressure position, pulse rate reduces. Beta wave frequency positively correlated with pulse rate at sitting blood pressure measurement position. Interestingly, there is a nexus between an increase in cortical activation and sympathetic activity [19]. During standing blood pressure measurement position, alpha and beta wave frequency did not correlate with pulse rate.

This study had a few limitations despite providing valuable insight into the clinical significance of blood pressure positional changes leading to an increase in visual cortical activities in subjects. The study had a small sample size. Only females were used for the study due to a lower orthostatic tolerance. Therefore, further research should be extended to cover all sexes and age groups for better generalizability.

## Conclusions

The results of the present study indicated that both sitting and standing blood pressure measurement positions caused a decrease in alpha wave frequency when compared with baseline, but the standing blood pressure measurement position elicited a lower alpha wave frequency when compared with the sitting blood pressure measurement position. Thus, discrepancies in blood pressure measurement caused by changes in brain activities can be identified and ruled out using Powerlab to ensure accurate diagnosis. Overall, this study translates the possibility of using polygraphs to identify and manage discrepancies in blood pressure measurement caused by posture-induced changes in brain activities for accurate diagnosis.
